# Exploring Gendered Relational Dynamics in Palliative Care: A Qualitative Study on Women Professional Caregivers in India

**DOI:** 10.7759/cureus.69984

**Published:** 2024-09-23

**Authors:** Priya Joseph, Akhil P Joseph, Anithamol Babu, L T Om Prakash

**Affiliations:** 1 Department of Sociology and Social Work, CHRIST, Bengaluru, IND; 2 School of Social Work, Marian College Kuttikkanam Autonomous, Kuttikkanam, IND; 3 School of Social Work, Tata Institute of Social Sciences Guwahati Off Campus, Jalukbari, IND

**Keywords:** caregivers, gendered dynamics, palliative care, resilience, women

## Abstract

Introduction: The gendered dynamics in palliative care settings in India are deeply influenced by societal norms, which position caregiving primarily as a woman's responsibility. This study explores the experiences of women caregivers in Bengaluru, Karnataka, India, to understand their challenges and strategies.

Methodology: This qualitative study used a narrative approach to investigate the lived experiences of 38 women caregivers from six palliative care institutions in Bengaluru. Semi-structured interviews conducted in Kannada captured the nuances of their caregiving roles. Thematic analysis was employed to identify key themes related to caregiving challenges and coping mechanisms.

Results: The study reveals that women caregivers face significant burdens due to societal expectations and institutional constraints, including resource shortages and limited professional development opportunities. Despite these challenges, the women demonstrated resilience through strong social networks, spiritual practices, and effective time management. However, relying on informal support systems highlights a gap in formal institutional support.

Implications: The findings emphasise the need for comprehensive support systems within palliative care institutions that address the practical and cultural challenges of caregiving. Future research should include a more diverse sample, including male caregivers, and explore the evolving nature of caregiving challenges.

## Introduction

India's societal landscape is marked by deep inequalities, with health inequality being particularly urgent in the context of the country's evolving social dynamics. These disparities arise from inadequate public health investment and broader systemic flaws, such as skewed developmental policies and narrowly conceived health strategies [1,2]. Within this framework, palliative care introduced in India in the 1960s remains underdeveloped and largely inaccessible, especially in rural and semi-urban areas. This inaccessibility is deeply intertwined with the broader socio-cultural shifts in India, where traditional family structures that once provided informal care are weakening [3]. As modern life imposes increasing demands, the need for formal palliative care services has become more pressing. However, in a society where gender inequality is deeply entrenched, the burden of caregiving-particularly for the terminally ill-falls disproportionately on women. Despite ongoing discussions on women's empowerment, cultural norms persist in framing caregiving as a female duty, reflecting societal neglect in addressing systemic inequalities, which further marginalizes and undervalues women in these roles [4,5].

Extensive literature highlights the gendered nature of caregiving in India, where women are expected to fulfil multiple caregiving roles, including child-rearing, food preparation, and caring for the elderly and sick [4,6,7]. The responsibility for caring for terminally ill patients, especially those in need of palliative care, is predominantly assigned to women, not by choice but through systemic impositions rooted in patriarchal traditions. These expectations significantly burden women, who often manage these responsibilities with minimal external support or recognition. The intersection of these cultural expectations with the increasing demands of contemporary life forces women to navigate complex caregiving roles, often at the cost of their well-being. This situation calls for a critical re-evaluation of traditional caregiving roles and societal expectations placed on women, particularly in palliative care [8,9].

This study investigates the gender dynamics of palliative care in India, focusing on the lived experiences of women professional caregivers. It explores how women perceive and navigate their caregiving roles within the socio-cultural framework of Indian society and the specific challenges and coping strategies they employ, particularly in urban settings like Bengaluru. The study aims to examine women's systemic vulnerabilities critically, understand the socio-cultural factors shaping these roles, and develop actionable recommendations for more equitable, community-based approaches to palliative care that fully recognise and support women’s vital contributions.

## Materials and methods

Research design

This qualitative study explored women's experiences in palliative care service delivery in urban Bengaluru, India, using a narrative design to capture their lived experiences, perspectives, and challenges. This approach was chosen because it can delve into the complex relational dynamics women caregivers navigate within palliative care institutions [10].

Setting and participants

The study was conducted across six diverse palliative care institutions in Bengaluru. The institutions were selected through purposive sampling, guided by the Pallium India directory, to ensure they represented diverse palliative care settings relevant to the study’s focus.

Participant selection

Participants included 38 women caregivers, selected based on inclusion criteria requiring active involvement in palliative care, either in professional or managerial roles, with at least one year of experience. The sample size was determined by the principle of data saturation, ensuring a comprehensive exploration of the gendered experiences and challenges these women face.

Data collection

Primary data were collected through semi-structured interviews to elicit detailed accounts of participants' roles, challenges, and experiences in palliative care. Interviews were conducted in Kannada, the local language, to capture authentic narratives and ensure cultural relevance. All interviews were audio-recorded with consent and transcribed verbatim for analysis. Secondary sources, such as reports and policy documents related to palliative care, were also reviewed to contextualise findings and enhance the overall analysis.

Data analysis

Data were analysed using the thematic analysis of Brown & Clerk, which involved systematically coding the transcribed interviews and organising these codes into overarching themes [11]. This iterative process allowed for continuous refinement to capture the complexities of the participants' experiences, with particular attention to challenges and coping strategies. The use of qualitative data analysis software ensured a robust and systematic analysis, and researcher bias was managed through reflexive practices and regular team discussions.

Ethical considerations

The study adhered to ethical standards, with the approval from the Departmental Ethical Review Board (DERB) for Social Work Research at Marian College Kuttikkanam Autonomous (MCKA/USW/SF/F/202), obtained prior to data collection. Informed consent was secured from all participants, ensuring they were fully aware of the study's purpose, risks, benefits, and rights, including the option to withdraw at any time. Confidentiality and anonymity were rigorously maintained, and potential ethical dilemmas were managed by providing participants with support resources and ensuring a culturally sensitive approach throughout the study [12].

## Results

Socio-demographic characteristics

The study involved 38 women caregivers from six diverse palliative care institutions in Bengaluru (summarised in Table 1). The participants were divided into two age groups: 25-35 years (17) and 36-45 years (21). In terms of educational qualifications, 15 participants held undergraduate degrees, while 23 had postgraduate qualifications. Occupations included medical professionals (five), nursing professionals (16), social workers (nine), and administrative staff (eight). All participants had been married for 5-25 years. In terms of children, eight had one child, 20 had two, and nine had three or four. Socio-economically, 12 were from lower, 20 from middle, and six from upper-middle backgrounds.

**Table 1:** Participants’ profile

Characteristics		Number
Age (Years)	25-35	17
	36-45	21
Education	Undergraduate degrees	15
	Postgraduate degrees	23
Occupation	Medical professionals	5
	Nursing professionals	16
	Social workers	9
	Administrative staff	8
Duration of marriage	5-25 years	38
Number of children	One	8
	Two	20
	Three or four	9
Socio-economic status	Lower	12
	Middle	20
	Upper-middle	6

The burden of gendered expectations

The first theme that emerged from the analysis was the significant burden of gendered expectations placed on women in palliative care settings (Table 2). The participants frequently discussed how societal and cultural norms dictated that caregiving was inherently a woman’s responsibility. This expectation extended beyond their professional duties, influencing their roles within their families and communities. Many women felt overwhelmed by the dual responsibilities of professional caregiving and managing their household duties, which included child-rearing, maintaining the household, and caring for elderly family members. These expectations were particularly pronounced among those with lower socio-economic status, who had fewer resources and support systems.

**Table 2:** The burden of gendered expectations

Theme	Sub-Theme	Participant Verbatim
The burden of gendered expectations	Societal and cultural norms dictating caregiving roles	"It is always assumed that caregiving is a woman's job, regardless of what else she might be doing."
	Dual responsibilities of professional and domestic caregiving	"I finish a full day at work, only to come home to another full set of responsibilities."
	Impact of socio-economic status on caregiving burdens	"We cannot afford any outside assistance, so I have to manage everything alone, which is overwhelming."

Coping strategies and resilience

The second theme identified was the coping strategies and resilience demonstrated by the participants in response to the demands of their caregiving roles (Table 3). Despite the heavy burdens, the participants demonstrated a range of coping strategies that enabled them to manage their roles effectively. Resilience was a recurring theme, with many women employing practical and emotional strategies to cope with the demands of their caregiving roles. Some participants relied on strong social networks, including support from colleagues, family members, and community groups. Others spoke of developing personal resilience through spiritual or religious practices, which provided them with emotional strength and a sense of purpose. Additionally, time management and prioritisation were frequently mentioned as participants juggled multiple responsibilities.

**Table 3:** Coping strategies and resilience

Theme	Sub-Theme	Participant Verbatim
Coping strategies and resilience	Utilisation of social support networks	"I lean on my family and colleagues a lot; without their support, I could not manage everything."
	Spiritual and religious practices for emotional resilience	"My faith keeps me going; prayer gives me the strength to face each day."
	Time management and prioritisation skills	"I have learned to prioritise and manage my time carefully, or else I would be overwhelmed."
	Emotional self-care and stress management	"Even just a few minutes of quiet time can make all the difference in how I handle the stress."

Professional identity and emotional labor

Another critical theme identified was the interplay between the women’s professional identities and the emotional labour involved in palliative care (Table 4). Many participants described their work as emotionally taxing but also deeply fulfilling. The emotional labour associated with providing care to terminally ill patients often extends beyond the workplace, affecting their mental and emotional well-being. The participants articulated a strong sense of duty and empathy, which they felt was essential to their role, but also acknowledged that it often led to burnout and emotional exhaustion. The participants revealed that women in administrative roles, while less involved in direct patient care, still experienced significant emotional labour, particularly in managing staff and addressing the challenges faced by the caregiving teams.

**Table 4:** Professional identity and emotional labour

Theme	Sub-Theme	Participant Verbatim
Professional identity and emotional labour	Emotional fulfilment	"Even though it is exhausting, I find a deep sense of fulfillment in knowing I am making a difference in my patients’ lives."
	Emotional overflow	"The emotional weight of what I do follows me home, and sometimes it feels overwhelming."
	Duty and empathy	"I cannot imagine doing this job without empathy; it drives me, even when completely drained."
	Burnout	"There are days when I feel completely burnt out, like I have nothing left to give, but I know I must keep going."
	Administrative emotional labor	"Even though I am not directly caring for patients, managing the staff and their challenges takes a huge emotional toll on me."

Institutional challenges

The participants also highlighted various institutional challenges that impacted their caregiving roles (Table 5). These challenges included inadequate resources, such as insufficient staff and medical supplies, often placing additional pressure on the caregivers. The women noted that these institutional constraints were exacerbated by the limited availability of training and professional development opportunities, particularly in specialised areas of palliative care. The institutional environment, including the hierarchical structure within these organisations, sometimes created barriers to effective communication and collaboration, further complicating the caregiving process.

**Table 5:** Institutional challenges

Theme	Sub-Theme	Participant Verbatim
Institutional challenges	Resource shortages	"We are constantly short on staff and supplies, which makes our job so much harder."
	Limited training opportunities	"There is hardly any chance to get specialised training, leaving us unprepared for complex cases."
	Lack of professional development	"I feel stuck in my role because there are hardly any opportunities to learn new skills or advance."
	Pressure from institutional constraints	"The expectations are high, and meeting them with available resources can be challenging."

Socio-economic influences on caregiving

Socio-economic status emerged as a significant theme influencing the participants' experiences (Table 6). Women from lower socio-economic backgrounds reported more significant difficulties in balancing their professional and personal responsibilities, often due to limited access to support services and financial resources. In contrast, those from middle and upper-middle-class backgrounds had slightly more access to external support, which eased some of the caregiving burdens. However, across all socio-economic groups, the strain of managing caregiving duties alongside financial concerns was a common theme, affecting the women’s overall well-being and their ability to provide high-quality care.

**Table 6:** Socio-economic influences on caregiving

Theme	Sub-Theme	Participant Verbatim
Socio-economic influences on caregiving	Financial struggles	"It is hard to manage everything on a tight budget, especially when there is no extra money for help."
	Limited support access	"We do not have the luxury of hiring outside help, so I have to handle everything independently."
	Balancing work and home	"Balancing work and home is draining, especially without the resources to ease the burden."
	Support through stability	"A little financial stability allows me to get some help, making things much more manageable.
	Financial stress across classes	"Even with better finances, the stress of managing caregiving and financial worries never really disappears."

Educational influence on caregiving

The educational influence on caregiving has emerged as a significant theme (Table 7). The participants' educational backgrounds played a crucial role in shaping their caregiving approaches and coping mechanisms. Women with postgraduate education often reported feeling more confident and equipped to handle the complex demands of palliative care, drawing on their formal training and knowledge. These participants were also more likely to engage in reflective practices and seek professional development opportunities to enhance their caregiving skills. In contrast, those with undergraduate degrees expressed a need for more training and support in palliative care, indicating a gap in their available resources.

**Table 7:** Educational influence on caregiving

Theme	Sub-Theme	Participant Verbatim
Educational influence on caregiving	Confidence from higher education	"My postgraduate training gives me the confidence to handle even the toughest situations in palliative care."
	Reflective practice	"I often reflect on my experiences to improve my caregiving skills, something I learned during my advanced studies."
	Seeking professional growth	"I am always seeking new training opportunities to stay updated and improve my caregiving."
	Training gaps for undergraduates	" I need more training to be effective in my role; what I learned in my undergraduate program is insufficient."

## Discussion

This study examines the gendered dynamics faced by women caregivers in palliative care settings in Bengaluru, India, uncovering significant challenges deeply rooted in entrenched societal and cultural norms. These norms persist in framing caregiving as primarily a female responsibility, forcing women to juggle both professional and domestic roles without adequate support [13]. This burden is particularly pronounced for women from lower socio-economic backgrounds [14,15]. Consistent with existing literature, these findings highlight the compounded pressures on women with limited resources and the need for interventions that address both the logistical challenges of caregiving and the cultural norms that reinforce gender inequality. Despite these challenges, participants demonstrated resilience through various coping strategies. Social support networks, spiritual practices, and effective time management were critical methods for managing caregiving demands [16,17]. The reliance on personal networks and religious practices highlights a vital need for formal support systems within palliative care institutions to address the comprehensive needs of patients [18,19]. This indicates the need for more structured support mechanisms to ensure caregivers are not solely dependent on informal systems for coping [20,21].

The study also highlights the dual nature of caregiving which participants found both rewarding and exhausting, with professional identity and emotional labour emerging as critical themes. Participants expressed fulfillment in their roles but noted significant burnout, notably where institutional support was lacking. Even those in administrative roles reported considerable emotional labor, indicating that this challenge is widespread across various caregiving positions [22,23]. Additionally, institutional challenges, such as resource shortages, limited training opportunities, and communication barriers due to hierarchical structures, exacerbated the difficulties of caregiving [24,25]. These issues align with existing studies showing how institutional constraints can increase stress and reduce job satisfaction among caregivers. The lack of professional development opportunities underscores the need for palliative care institutions to invest in training and support for their staff [26].

Socio-economic status played a pivotal role in shaping participants' experiences, with women from lower socio-economic backgrounds facing disproportionately more significant challenges in balancing their responsibilities, highlighting stark socio-economic disparities [27]. Although financial stability can alleviate some burdens, stress related to managing work and financial concerns persists across all socio-economic groups. This highlights the need for policies that provide financial support and resources to caregivers, particularly those from disadvantaged backgrounds, to ensure they can fulfil their roles effectively. Educational background further shaped caregiving approaches. Women with postgraduate education felt more confident and better equipped to handle palliative care demands, often engaging in reflective practices and seeking professional growth opportunities [28]. In contrast, undergraduate graduates needed more training and support, revealing a resource gap for less-educated caregivers. This suggests that palliative care institutions should provide equitable support and training opportunities for all caregivers, regardless of their educational background [29,30].

While this study provides critical insights into the gendered dynamics of caregiving in palliative care settings in Bengaluru, several limitations must be acknowledged. The relatively small and homogenous sample, mainly composed of women with higher education levels, may not fully capture the diverse experiences of caregivers across different socio-economic backgrounds or regions. The exclusive focus on female caregivers also limits the ability to compare their experiences with male caregivers. Additionally, the reliance on self-reported data introduces potential biases, and the study's cross-sectional nature only provides a snapshot in time, missing the evolving nature of caregiving challenges. Future research should be broader to deepen our understanding of these issues.

## Conclusions

This study highlights the significant gendered challenges faced by women caregivers in Bengaluru's palliative care settings, where societal norms position caregiving as a female responsibility. Women, particularly those from lower socio-economic backgrounds, struggle to balance professional and domestic roles with limited institutional support, relying heavily on informal networks for resilience. The findings underscore the profound impact of gendered expectations, professional identity, and institutional challenges on these caregivers' well-being. However, the study's small sample size and focus on six institutions limit the generalizability of the findings. Future research should expand the sample size, explore the long-term effects of caregiving on women's health, and include male caregivers to understand gender dynamics in palliative care better.
